# Playing the pipes: acoustic sensing and machine learning for performance feedback during endotracheal intubation simulation

**DOI:** 10.3389/frobt.2023.1218174

**Published:** 2023-10-30

**Authors:** Torjus L. Steffensen, Barge Bartnes, Maja L. Fuglstad, Marius Auflem, Martin Steinert

**Affiliations:** ^1^ Department of Circulation and Medical Imaging, Norwegian University of Science and Technology, Trondheim, Norway; ^2^ Department of Mechanical Engineering, Norwegian University of Science and Technology, Trondheim, Norway

**Keywords:** medical simulation training, endotracheal intubation, acoustic sensing, machine learning in healthcare, convolutional neural network (CNN), support vector machine (SVM), audio classification, transfer learning

## Abstract

**Objective:** In emergency medicine, airway management is a core skill that includes endotracheal intubation (ETI), a common technique that can result in ineffective ventilation and laryngotracheal injury if executed incorrectly. We present a method for automatically generating performance feedback during ETI simulator training, potentially augmenting training outcomes on robotic simulators.

**Method:** Electret microphones recorded ultrasonic echoes pulsed through the complex geometry of a simulated airway during ETI performed on a full-size patient simulator. As the endotracheal tube is inserted deeper and the cuff is inflated, the resulting changes in geometry are reflected in the recorded signal. We trained machine learning models to classify 240 intubations distributed equally between six conditions: three insertion depths and two cuff inflation states. The best performing models were cross validated in a leave-one-subject-out scheme.

**Results:** Best performance was achieved by transfer learning with a convolutional neural network pre-trained for sound classification, reaching global accuracy above 98% on 1-second-long audio test samples. A support vector machine trained on different features achieved a median accuracy of 85% on the full label set and 97% on a reduced label set of tube depth only.

**Significance:** This proof-of-concept study demonstrates a method of measuring qualitative performance criteria during simulated ETI in a relatively simple way that does not damage ecological validity of the simulated anatomy. As traditional sonar is hampered by geometrical complexity compounded by the introduced equipment in ETI, the accuracy of machine learning methods in this confined design space enables application in other invasive procedures. By enabling better interaction between the human user and the robotic simulator, this approach could improve training experiences and outcomes in medical simulation for ETI as well as many other invasive clinical procedures.

## 1 Introduction

Airway management is critically important in emergency medicine and critical care (Trueger, 2013). Endotracheal intubation (ETI) is a widely used technique in intensive care units (Narendra et al., 2016) for unconscious or semi-conscious patients who cannot maintain breathing independently (Trueger, 2013; Kovacs and Sowers, 2018). This invasive non-surgical procedure involves the insertion of an endotracheal tube to establish an artificial airway, securing the airway and preventing aspiration while allowing healthcare practitioners to control ventilation.

However, ETI carries risks, such as laryngotracheal injury (Tikka and Hilmi, 2019), and hypoxia or even brain damage and death if a free airway is not established in critical care settings. Consequently, adequate training in laryngoscopy and ETI is crucial for safe and accurate procedural execution. Patient simulators play an important role in this training, complementing procedural competence and failure mode awareness before practicing on real patients.

In simulation-based training for clinical skills and procedural routines, such as ETI, the equipment should demand similar cognitive and psychomotor loading as performing the routines would on real patients (McGaghie et al., 2006). This is considered by the ecological validity of simulation, meaning that the simulated procedure should facilitate repeated practice of relevant tasks by using sufficient equipment for trainees to realistically perform them (Kushniruk et al., 2013). The design of simulators should, therefore, provide lifelike experiences for both skilled and novice medical practitioners in training, which is essential to mitigate sources of false learning and achieve familiarity effects for skills and experiences to transfer into clinical practice. Replicating relevant anatomy accurately is often complex, yet critical for practitioners to develop the skills and knowledge required to perform procedures such as ETI safely.

Providing actionable and descriptive feedback during and after training events is paramount (Issenberg et al., 2005; McGaghie et al., 2006). Quality performance metrics and feedback should integrate within and enhance simulated training models, crucially without compromising simulator realism. This would require sensors or other foreign elements to not cause visual or tactile disturbances, limiting training validity. Replicating the tactile sensation of ETI while providing performance feedback is a significant challenge in ETI simulation; a design objective with anatomical constraints, tactile compliance requirements, and obstructed visibility of the procedure.

This paper presents a proof of concept of a method that combines acoustic measurement and artificial intelligence (AI) to classify ETI user performance on patient simulators in terms of endotracheal intubation depth and cuff inflation status. This method transmits inaudible ultrasonic pulses into the simulated manikin airway and records the sound in a location away from the trachea. As the airway geometry is highly complex, traditional sonar methods are difficult to implement without sacrificing anatomical accuracy. Instead, machine learning classifiers are trained on the reflected audio’s spectral content to detect subtle changes in reflected sound resulting from altered airway geometry acoustics. The method is non-intrusive and avoids tracheal instrumentation that could interfere with procedure realism.

To demonstrate the feasibility of this proof of concept, expert users performed 240 intubations on a patient simulator to generate a dataset for training and validating our model. To investigate the effect of equipment location, two configurations were used for both the sound source and microphone. Two classification approaches were employed: support vector machine (SVM) and convolutional neural network (CNN) with transfer learning. By using two different approaches, we aimed to enhance the robustness of our findings and prompt deeper understanding of the integration problem as well as underlying patterns in the data. SVM provides a solid baseline for audio classification performance (Cortes and Vapnik, 1995; Laput et al., 2015), while CNN transfer learning, leveraging pre-trained models, captures more complex relationships within the data, leading to potentially better performance (Pan and Yang, 2010; LeCun et al., 2015). The best-performing model was tested using a leave-one-intubation-out cross-validation scheme, achieving a median best performance of 98% global accuracy on 1-s audio recordings.

This approach provides a valuable tool for classifying endotracheal intubation training performance on patient simulators. The system is flexible and easily integrated, with potential applications beyond intubation training. By demonstrating the success of this initial proof of concept, we hope to enable integration in medical simulation, where providing quality performance metrics and feedback can support better training experiences and outcome.

## 2 Materials and methods

### 2.1 Intubation procedure

Intubation is a crucial procedure in airway management, involving the administration of anesthesia and muscle relaxants to the patient, followed by preoxygenation with 100% oxygen via a mask and bag (Butterworth et al., 2013; Wiengand, 2016). The endotracheal tube is then inserted using a laryngoscope to expose the vocal cords, with the tube’s depth indicated by markings on the tube (Haas et al., 2014). Once the tube is in place, the cuff is inflated sufficiently to ensure a proper seal and prevent movement during the procedure (Artime and Hagberg, 2020; Hagberg, 2022). The endotracheal tube’s cuff serves two main purposes: preventing gastric content and other substances from entering the airways and avoiding airflow leakages around the tube (Nejla et al., 2017).

Incorrect cuff inflation can lead to complications. Underinflation may result in microaspiration (Stewart et al., 2003; Gunasekera and Gratrix, 2016; Nejla et al., 2017) or laryngeal and tracheal complications due to tube movement (Jougon et al., 2000). Overinflation can cause tracheal mucosal damage (Tu et al., 1999; Gunasekera and Gratrix, 2016), ischemia, ulceration, inflammation, tracheal rupture, granulation, and tracheal stenosis (Ganner, 2001; Fan et al., 2004; Butterworth et al., 2013).

Proper endotracheal tube placement minimizes risk of airway injury (Mort et al., 2013). Shallow placement can damage the upper airway structures, including vocal cord paralysis, ulceration, or dysfunction (Lu et al., 1999; Butterworth et al., 2013; Mort et al., 2013). Deep placement can impact the carina, leading to hypertension, tachycardia, and bronchospasm (Varshney et al., 2011). Moreover, endobronchial intubation, or unintended placement of the tube in the left or (more commonly) right main bronchi, can result in one-lung ventilation (De Marchi, 2014; Lohser and Slinger, 2015; Heyne et al., 2022), causing injury to both lungs. The ventilated lung can suffer from tension pneumothorax (Salem and Baraka, 2013) and reduced residual air (Lohser and Slinger, 2015), while the unventilated lung may experience atelectasis, hypoxemia, and hypercapnia (Lohser and Slinger, 2015).

### 2.2 Data acquisition

#### 2.2.1 Acoustic equipment

A full-size patient simulator (SimMan, Laerdal Medical, Norway) was outfitted with two small electret condenser microphones (EM272, Primo, Japan) and a small piezoelectric buzzer (MCUST10P40B07RO, Farnell, United States). The microphones are small enough to be built into space-constrained environments and are sensitive to ultrasonic frequencies. The usable frequency range extends to approximately 60 kHz, but with strong attenuation above approximately 40 kHz. The two mono microphones were connected in stereo to an audio A/D interface (Xonar U5, ASUS, Taiwan) and recorded at a sample rate of 192 kHz to a MacBook as separate audio channels using open-source software (Audacity 3.2.5, Audacity Team).

The piezo buzzer has a peak sound pressure at 40 kHz, but also a reasonable output from about 10 kHz to 60 kHz. During the experiment, the buzzer was driven using an arbitrary waveform generator (UTG 2025A, Uni-Trend, China). A few different waveforms were tried, but in the end we followed the suggestion of earlier work (Laput et al., 2015) and used a repeating sinusoidal linear sweep from 20 kHz to 60 kHz with a sweep duration of 100 m. The resulting output was inaudible and seemed to provide good classification performance in our setting.

**FIGURE 1:**
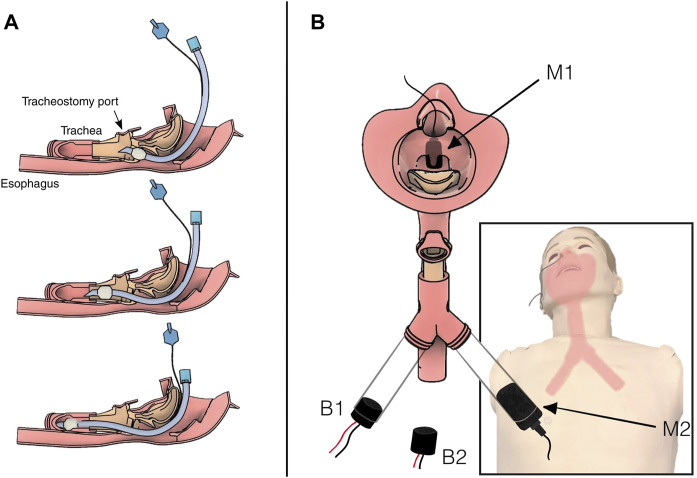
Diagram of the airway showing intubation tube placement and instrumentation. **(A)** Three progressively deep intubations in the simulated airway. Top: shallow intubation. The tube has correctly entered the trachea but at insufficient depth. Middle: correct placement deep into the trachea, but not at risk of impacting the carina. Bottom: deep intubation. The tip of the tube is hitting the carina, and the tube is on its way into the right bronchus. **(B)** Illustration of the location of the two microphones, M1 above the airway in the nasal cavity and M2 below the airway in the left bronchus. The two buzzer configurations, B1 inside the right bronchus and B2 outside the airway in the chest cavity. Note that B1 was not in place when B2 was active, and *vice versa*.

One microphone was fixed in the manikin’s head, above the airway, while the other was fixed in a tube below the airway (Figure 1). Two series of recordings were made. In the first series, the piezo buzzer was placed in the chest cavity of the manikin, outside the airway assembly, while in the second series, the buzzer was fixed directly in the airway tube across from the second microphone.

#### 2.2.2 Recording protocol

Intubations were recorded in six distinct categories derived from the combination of tube placement depth (correct, deep, and shallow) and cuff inflation status (inflated/deflated).

Prior to recording, an expert user performed the intubation procedure using a laryngoscope and standard ET tube. Following manikin manufacturer recommendations, a purpose-made silicone lubricant was applied before intubating. With the endotracheal tube in place, 25 s of audio were recorded while the buzzer was active. The tube’s cuff was then inflated, and another 25 s of audio were recorded.

In both series, 40 intubations were made in each of the three positions (shallow, correct, deep), resulting in 120 intubations per buzzer configuration and 240 intubations in total. With and without inflation, the total recorded data set consists of 480 recordings, divided equally among the six classes. A total of 12,000 s of audio were recorded (Table 1).

**TABLE 1:** Data breakdown.

Class	Recordings	Seconds
Shallow	80	2000
Shallow, inflated cuff	80	2000
Correct	80	2000
Correct, inflated cuff	80	2000
Deep	80	2000
Deep, inflated cuff	80	2000

### 2.3 SVM model

#### 2.3.1 Preprocessing and feature extraction

SVMs are robust models for complex ultrasound sound characterization (Temko and Nadeu, 2006; Laput et al., 2015). To train the SVM classifier, recordings from configuration B1, M2 (Figure 1) were first split into testing (20%) and training cohorts. Audio was highpass filtered using a Butterworth filter with a 20 kHz passband, emphasizing the inaudible portion of the spectrum, and normalized before being split into 0.5-second-long segments resulting in a total of 9,600 training and 2,400 test segments. For each segment, we extracted a set of features consisting of the zero-crossing rate, 2-norm, root-mean-square values, spectral centroids as well as the magnitude of the discrete FFT. The dimensionality of the resulting feature vector was reduced via principal component analysis. The first 1,000 components were kept, containing more than 99.9% of the explained data variance.

Features including mel-frequency cepstral coefficients and wavelet scattering coefficients resulted in worse performance (Supplementary Figure S1).

#### 2.3.2 Model training

A linear kernel SVM classifier with one vs. rest decision function was trained on the training data with two sets of labels using libsvm (Chang and Lin, 2011; Pedregosa et al., 2011). In the first label set, audio segments were only labeled according to depth of tube insertion, making three classes. In the second set, segments were additionally labelled with cuff inflation status, making six classes.

### 2.4 Transfer learning

In addition to the SVM classifier we employed a transfer learning approach using a pre-trained deep learning model, YAMNet (Hershey et al., 2017). YAMNet is a convolutional neural network (CNN) trained on the Audioset-YouTube corpus of labeled audio signals (Gemmeke et al., 2017) designed for relatively low-memory environmental sound classification. YAMNet has been demonstrated to be effective in transfer learning applications (Tsalera et al., 2021).

#### 2.4.1 Preprocessing

We processed the audio data similarly to the SVM model. Audio was highpass filtered with a 20 kHz passband, normalized, and split into 1-s chunks. A sliding-window approach could yield more spectrograms, but we opted not to do so in order to simplify input size analysis, additionally assuming the audio signal to be relatively stationary in our controlled environment.

**FIGURE 2:**
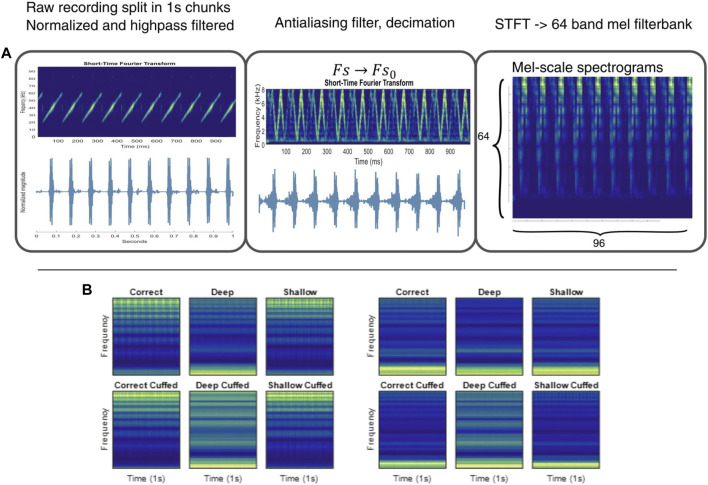
Illustration of YAMnet preprocessing and resulting spectrograms. **(A)**: A step-by-step depiction of the preprocessing pipeline, including audio segmentation, normalization, highpass filtering with an 18 kHz passband Butterworth filter, resampling to a 16 kHz sample rate, computing the STFT, and applying a 64-band mel scale filterbank to generate input spectrograms with 96 frames of 10 m duration. **(B)**: Comparison of mean spectrograms from 1,000 samples per case, recorded in the lower airway. Left: Buzzer located inside the airway assembly; Right: Buzzer placed in the chest cavity. The input features exhibit distinct qualitative differences.

The signal was resampled to 16 kHz using the MATLAB Signal Processing Toolbox. The short-time Fourier transform (STFT) was computed for each downsampled signal using a Hann window with period 128, FFT length of 512% and 75% overlap. The STFT was filtered with a 64-band mel-scale filter bank and the resulting spectrograms divided into 10 m bins, yielding 96 × 64 sized spectrograms suitable for input to the network (Figure 2).

#### 2.4.2 Model training

The pretrained model was loaded and trained using the Matlab Deep Learning Toolbox. The last fully connected layer and the output layer of the network were replaced to Connect a 6-label classification output layer. We did not freeze layers. Models were trained on a single GPU (GTX 2080 TI, Nvidia, United States) from a Windows computer with 16 GB RAM and a 1.7 GHz CPU, using the adam optimizer with mini batches of size 128 for 5 epochs and an initial learning rate of 0.001 halving every two epochs. The loss function was cross-entropy.

Training time was kept low as many models needed to be trained for our cross-validation scheme. 5 epochs was decided by observing that models tended to converge in that time scale.

#### 2.4.3 CNN Cross-validation

We implemented a leave-one-intubation-out cross-validation approach. One intubation (both deflated and inflated cuff) was excluded from the training process at a time. For each intubation, we trained the model on the remaining 239 intubations, and tested the classifier on the held-out intubation. Training one model this way took approximately 90 s, or about 6 h for all 240 intubations.

To compare the influence of microphone and buzzer location, we repeated the training and testing process for each of the four microphone and buzzer combinations: microphone over/under airway (M1, M2), and buzzer inside/outside the airway (B1, B2) (Figure 1). Total training time was thus about 24 h.

**FIGURE 3:**
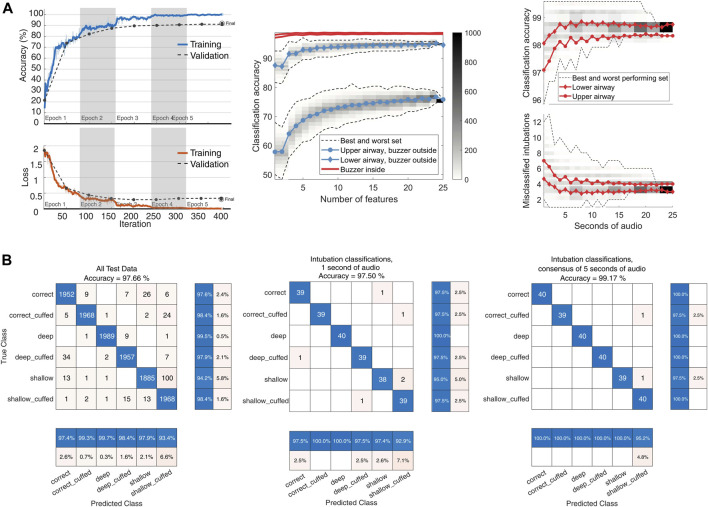
YAMNet classifier performance. **(A)**: left: model training progress from a model representative of the LOTO model training, with an added 15% validation split. Center: classifier input size sensitivity. Audio from 480 recordings each with 25 s of available audio was divided into 0.96-s segments. For input lengths ranging from 1 to 25 s, 1,000 random permutations of test features were used for classification. The class was determined by the mode of the classifications. The figure shows global accuracy for the four combinations of microphone and buzzer locations. Dashed lines represent the range of classification accuracy for each set size (*n* = 1–25), with color-coded histogram bins displaying the distribution of the 1,000 permutations per set size. The central line indicates the mean accuracy per set size. Right: Global accuracy and absolute number of misclassifications for the two top performing configurations. **(B)**: left: Center: Confusion matrix for the YAMNet classifier on the full feature set of 12,000 s of recordings. Center: Classifier performance when tested on a random 1-s segment from each intubation. Right: Classification using the mode of 5 one-second segments from each intubation.

To evaluate the classifier’s performance using different audio input sizes, we selected 1,000 random permutations of the 25 labelled 0.96-s spectrograms within each test intubation set. These permutations were incremented by 1 s, from 1 to 25 s. The output class was determined by the mode of the subset of test labels (Figure 3).

## 3 Results

### 3.1 SVM model

**FIGURE 4:**
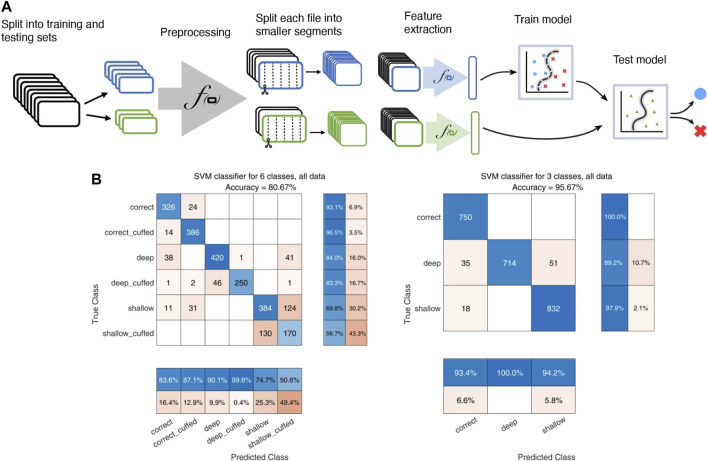
Illustration of the SVM model pipeline. **(A)**: Flowchart illustrating the SVM pipeline, including splitting of recordings into training and testing cohorts, the preprocessing step of highpass filtering with a 20 kHz Butterworth filter and normalization, segmenting into 0.5-s intervals, and extraction of various audio features for input to the classifier. **(B)**: confusion matrices for the SVM classifier. Left: classification using the full 6-label set of both depth and cuff status. Right: classification on the reduced-size 3 label set of only tube insertion depth.

For 100 random test-train splits the median global accuracy was 0.85 (0.70–0.93) on all 6 labels and 0.97 (0.91–0.99) for the reduced depth-only set of 3 labels. Confusion matrices for one test-train split using data from buzzer configuration 1 and microphone configuration 2 are shown in Figure 4. While the model has good sensitivity to intubation depth, it seems to have difficulty distinguishing cuff status. Sensitivity to cuff status during shallow intubations is particularly poor.

### 3.2 CNN model

The transfer learning approach using the pretrained YAMNet network enhanced performance on the audio classification task compared to the SVM classifier. Cross-validation results demonstrated high classification metrics across all categories, with macro averages for precision at 0.980. Performance metrics of the CNN model for the best audio configuration (B1, M2) are presented in Table 2. The classifier achieved a best global accuracy of 97.57% across the entire feature set of spectrograms (Figure 3). Per intubation, the trained classifier achieved a best global accuracy of 98.75% when exposed to the full set of 25 s of audio for each intubation case during the classification step. Best case accuracy reached 100% but went back down as classification set size approached the full classification set, reflecting the unbalanced distribution of incorrect predictions. Classification performance improved when pooling longer inputs (Figure 3). For the best-performing configuration, mean accuracy did not increase meaningfully with input sizes above 5 s of audio, although the worst-case classification accuracy did continue to improve until about 15 s.

**TABLE 2:** Classification metrics.

Metric	Correct	Correct cuffed	Deep	Deep cuffed	Shallow	Shallow cuffed	Macro AVG
True positive	974	975	999	985	960	984	980
False positive	16	2	0	9	26	70	21
False negative	26	25	1	15	40	16	21
True negative	4984	4998	5000	4991	4974	4930	4980
Precision	0,984	0,998	1,000	0,991	0,974	0,934	0,980
Sensitivity	0,974	0,975	0,999	0,985	0,960	0,984	0,980
Specificity	0,997	1,000	1,000	0,998	0,995	0,986	0,996
Accuracy	0,980	0,980	0,980	0,980	0,980	0,980	0,980
F−measure	0,979	0,986	0,999	0,988	0,967	0,958	0,980

### 3.3 Inference time

Preprocessing and inference time for 1-second-long segments of audio were timed using a Windows laptop with a 1.7 GHz CPU and 16 GB RAM. 100,000 segments were preprocessed and classified by a trained network, resulting in a combined median execution time of 27 m (IQR 2 m). Model load time was not considered.

### 3.4 Equipment configuration

Microphone and buzzer location had a large effect on the classifier’s performance. Microphone location 2 (inside the closed airway tubing) resulted in a consistently better classification accuracy. Microphone location 1 (mouth assembly) exhibited much worse classification accuracy when the buzzer was not placed inside the airway assembly, but only had slightly decreased performance with the buzzer in the airway.

## 4 Discussion

### 4.1 Equipment configuration and model performance

Our results demonstrated that the transfer learning approach using the pretrained YAMNet network outperformed the SVM classifier, with high classification metrics across all categories. Our findings indicate that the transfer learning strategy is effective in accurately classifying audio signals for this application, while the good SVM performance on the depth-only label set argues against the performance of the CNN model being due to overtraining or model-specific behaviour.

The equipment configuration (microphone and buzzer location) influenced classification performance. The best performance was achieved with microphone location 2, inside the closed airway tubing, and buzzer position 1, inside the opposing tube. This may be explained by the increased proximity of the buzzer to the microphone in this configuration. Sound recorded from the bronchial pathway could more reliably differentiate between changing windpipe geometries than the microphone above the airway, even with the buzzer outside the windpipe assembly. The difference between microphone locations was small in the “internal” buzzer configuration.

The pretrained CNN’s ability to classify intentionally inaudible sound events resampled from a much higher frequency range than originally trained on is noteworthy.

### 4.2 Comparison of SVM and CNN models

The pretrained CNN model outperformed the SVM model. This could potentially be explained by a CNN’s particular aptitude to learn complex patterns in high-dimensional data like audio signals (LeCun et al., 2015). The differences in performance between the two models could also be partially attributed to the relative complexity of the feature extraction process and interpretation. Future work on this approach could investigate the impact of using alternative or combined feature extraction techniques on SVM model performance.

### 4.3 Relation to other work

Using sound classification on ultrasound recordings to detect internal geometries has been demonstrated before, see for example Laput et al. (2015) where a SVM was trained to determine the geometry of interactive structures as an interactive device. Ultrasound classification has furthermore seen wide adoption in various human-computer interaction applications, including tactile user interfaces (Dahl et al., 2014).

While YAMNet has been used in many transfer learning applications for audio classification (Tsalera et al., 2021) its use on ultrasonic signals is not widely adopted. Ghani et al. (2023) presented an application of acoustic bird classification including ultrasonic bat calls among other higher frequency signals. They discuss techniques for transfer learning on ultrasonic signals using models trained on lower sample rates, such as pitch shifting, or in one case, simply ignoring the problem. They report reasonable results, although worse than purpose-built models. By comparison, we hypothesize that our good results using YAMNet is probably in no small part due to the controlled signal we create, which is likely to be much more distinct than bird calls recorded in the wild. Our controlled environment also allowed us to collect a larger dataset.

In the context of endotracheal tube location in physical patient simulators, previous approaches have included magnetic detection using Hall effect sensors outside the esophagus and magnets fixed to a modified endotracheal tube (Samosky et al., 2011) or pressure sensors inside modified manikin airways (Sprick et al., 2011). These techniques involve modifying the geometry of the airway or the endotracheal tube, which is undesirable as it has the potential to negatively affect training realism. Another approach has been confirmation of placement manually using disposable fiber-optic bronchoscopes inserted through the ET tube (Mitra et al., 2019). While this latter method was reported to be effective, we assess that automated placement confirmation is desirable with no modification to ET equipment or the critical geometry of the airway.

### 4.4 Network activation

**FIGURE 5:**
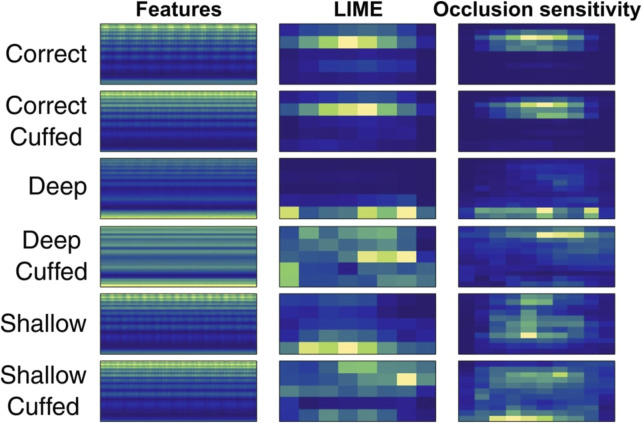
LIME and occlusion sensitivity maps across 1,000 averaged input features in the best performing configuration.

Given the significant compression to a low sampling rate, it is prudent to examine the features of the compressed signal on which the CNN operates. Figure 5 presents averaged spectrograms for each condition with LIME and occlusion sensitivity maps. LIME (locally interpretable model-agnostic explanations) and occlusion sensitivity are decision explanation tools that assign a relative importance to regions of the input data (Kakogeorgiou and Karantzalos, 2021). These maps help estimate the input data’s relative importance for classification decisions. Notably, even with very aggressive compression and aliasing, the higher frequency range remained active, particularly for the “correct” condition. However, classification performance for shallow intubation was consistently lower, possibly due to the lack of distinct discriminatory feature regions in this condition.

### 4.5 Advantages of acoustic sensing in medical simulators

The remote nature of acoustic sensing offers flexibility for designers, allowing them to instrument simulators without altering the relevant anatomy or compromising the fidelity or validity of the simulation. As a “black box” solution, our approach requires minimal implementation effort, and to our knowledge no comparable AI-based acoustic classification methods have been reported to similar ends in medical simulators. Although our proof of concept focused on intubation training, ease of integration implies it could be extended to other procedures where the introduction of instruments into the patient’s body is involved, such as larynx mask insertion, phlegm suction, and intravascular procedures.

### 4.6 Implications for designers and training outcomes

Our acoustic sensing approach offers designers flexibility and ease of integration, enabling them to incorporate advanced sensing into existing product architectures without altering simulated anatomical structures. While we cannot draw definitive conclusions about the impact on medical training outcomes, improving the quality of simulator feedback could potentially enhance them. By providing insights into otherwise invisible processes, our approach can improve feedback and assessment, supporting better training experiences and outcomes in medical simulation.

In a training scenario, the proposed classification system must produce a decision in a time-constrained setting. Given our median inference time of 27 m, a total execution time for a 5-s inference of around 100–200 m may be acceptable for real-time evaluation, depending on the goal of the implementation. For a training feedback scenario, either intermittently live during performance or in a post-scenario debrief setting, time constraints may be much looser, for example.

The hardware we used for timing is comparable to that available in a live simulation using the patient simulator described in this work. However, YAMNet models have been used for real-time sound event detection using low-resource edge devices such as the Raspberry Pi 4 (Mesa-Cantillo et al., 2023). With concessions to the higher audio sample rate required for ultrasonic applications, future applications could be deployed to environments with fewer computational resources available, for example simpler training devices with fewer resources available.

### 4.7 Model design

The proposed feedback model can only provide feedback on discrete states, not a continuous measurement of insertion depth. Discrete state classification was chosen due to practical relevance, reduced complexity, and increased robustness. Discrete classification aligns with intubation training goals, as it focuses on distinguishing correct from incorrect tube placement. This approach simplifies the task of the feedback system, avoiding challenges associated with the complex airway geometry. Moreover, discrete classification is likely to be more robust and less sensitive to measurement noise. It may more easily be generalizable across different training scenarios where this approach may be relevant.

### 4.8 Study limitations and future work

The study was conducted in a controlled setting, which may not accurately represent the variability and challenges encountered in real-world medical training environments. Factors such as background noise, differences in equipment, and variations in patient simulators might impact the performance of our acoustic sensing method. Secondly, the evaluation of preprocessing methods and SVM features was not exhaustive. Although our approach demonstrated promising results using the YAMNet network and transfer learning, other preprocessing steps and classification algorithms might yield better performance on more explainable models and might be more robust outside a controlled lab setup.

Additionally, our study did not account for potential confounders such as microphone and buzzer calibration. The performance of the acoustic sensing method could be influenced by inconsistencies in the hardware or differences in the acoustics of the training environment. Future research could evaluate the robustness and generalizability of this acoustic sensing approach using different equipment and less controlled environments.

## 5 Conclusion

Acoustic sensing in combination with machine learning can capture geometric changes in complex anatomical structures with high accuracy. This enables collecting valuable feedback on otherwise hidden processes during skill and procedural training on simulated anatomy. This study highlights the value of the acoustic sensing approach in providing a simple and effective solution for classifying ETI user performance. Remarkably, the method achieved good results even without serious attempts at equipment calibration or an exhaustive evaluation of features or algorithms. This robustness lowers implementation cost and adaptability for use in various clinical training contexts. Reasonable classification performance on a secondary machine learning model trained on different feature types on the same data set is encouraging, indicating that the high performance of the CNN model is not the result of overfitting.

Our results highlight the effect of microphone and buzzer positioning on classifier performance, and the significance of optimal placement to achieve accurate results. We further illustrate the positive correlation between increased test input size and classifier accuracy, indicating the potential benefits of utilizing longer audio segments for improved classification reliability.

By refining and building upon this initial work, similar acoustic sensing methods could become a valuable tool for enhancing the quality of feedback and performance metrics in medical simulation, contributing to better training experiences and outcomes in critical airway management procedures. Further investigation may focus on refining the models and examining the potential integration of this approach into real-time monitoring systems for intubation training procedures.

## Data Availability

The datasets presented in this study can be found in online repositories. The names of the repository/repositories and accession number(s) can be found below: Zenodo: doi.org/10.5281/zenodo.7902735.

## References

[B1] Artime A. Hagberg C. A. (2020). Airway management in the adult. *Miller’s Anesthesia* . 9th ed. Philadelphia: Elsevier, 1373–1412.

[B2] Butterworth J. F. Mackey D. C. Wasnick J. D. (2013). Morgan & mikhail’s clinical anesthesiology. 5th ed McGraw Hill Education, New York City, NY, United States. McGraw-Hill Education.

[B3] Chang C. C. Lin C. J. (2011). LIBSVM: a library for support vector machines. ACM Trans. Intell. Syst. Technol. 2 (27), 1–27. 10.1145/1961189.1961199

[B4] Cortes C. Vapnik V. (1995). Support-vector networks. Mach. Learn. 20, 273–297. 10.1023/A:1022627411411

[B5] Dahl T. Ealo J. L. Bang H. J. Holm S. Khuri-Yakub P. (2014). Applications of airborne ultrasound in human–computer interaction. Ultrasonics 54, 1912–1921. 10.1016/j.ultras.2014.04.008 24974162

[B6] De Marchi L. (2014). “Endobronchial intubation,” in Anesthesiology core review: Part One basic exam. Editors Freeman B. S. Berger J. S. (New York, NY: McGraw-Hill Education). Available at: accessanesthesiology.mhmedical.com/content.aspx?aid=1102567869 (Accessed April 19, 2023).

[B7] Fan C. M. Ko P. C. I. Tsai K. C. Chiang W. C. Chang Y. C. Chen W. J. (2004). Tracheal rupture complicating emergent endotracheal intubation. Am. J. Emerg. Med. 22, 289–293. 10.1016/j.ajem.2004.04.012 15258871

[B8] Ganner C. (2001). The accurate measurement of endotracheal tube cuff pressures. Br. J. Nurs. 10, 1127–1134. 10.12968/bjon.2001.10.17.9952 11904572

[B9] Gemmeke J. F. Ellis D. P. W. Freedman D. Jansen A. Lawrence W. Moore R. C. (2017). “Audio Set: an ontology and human-labeled dataset for audio events,” in 2017 IEEE International Conference on Acoustics, Speech and Signal Processing (ICASSP), New Orleans, LA, USA, 05-09 March 2017 (IEEE), 776–780. 10.1109/ICASSP.2017.7952261

[B10] Ghani B. Denton T. Kahl S. Klinck H. (2023). Feature embeddings from large-scale acoustic bird classifiers enable few-shot transfer learning. arXiv. 10.48550/arXiv.2307.06292

[B11] Gunasekera P. Gratrix A. (2016). Ventilator-associated pneumonia. BJA Educ. 16, 198–202. 10.1093/bjaed/mkv046

[B12] Haas C. F. Eakin R. M. Konkle M. A. Blank R. (2014). Endotracheal tubes: old and NewDiscussion. Respir. Care 59, 933–955. 10.4187/respcare.02868 24891200

[B13] Hagberg C. A. (2022). Hagberg and benumof’s airway management Saunders. Philadelphia, Pennsylvania, United States. Elsevier Health Sciences. Available at: https://books.google.no/books?id=TiPuzgEACAAJ .

[B14] Hershey S. Chaudhuri S. Ellis D. P. W. Gemmeke J. F. Jansen A. Moore R. C. (2017). “CNN architectures for large-scale audio classification,” in 2017 IEEE International Conference on Acoustics, Speech and Signal Processing (ICASSP), New Orleans, LA, USA, 05-09 March 2017 (IEEE), 131–135. 10.1109/ICASSP.2017.7952132

[B15] Heyne G. Ewens S. Kirsten H. Fakler J. K. M. Özkurtul O. Hempel G. (2022). Risk factors and outcomes of unrecognised endobronchial intubation in major trauma patients. Emerg. Med. J. 39, 534–539. 10.1136/emermed-2021-211786 34376465 PMC9234407

[B16] Issenberg S. B. Mcgaghie W. C. Petrusa E. R. Gordon D. L. Scalese R. J. (2005). Features and uses of high-fidelity medical simulations that lead to effective learning: a BEME systematic review. Med. Teach. 27, 10–28. 10.1080/01421590500046924 16147767

[B17] Jougon J. Ballester M. Choukroun E. Dubrez J. Reboul G. Velly J. F. (2000). Conservative treatment for postintubation tracheobronchial rupture. Ann. Thorac. Surg. 69, 216–220. 10.1016/S0003-4975(99)01129-7 10654516

[B18] Kakogeorgiou I. Karantzalos K. (2021). Evaluating explainable artificial intelligence methods for multi-label deep learning classification tasks in remote sensing. Int. J. Appl. Earth Observation Geoinformation 103, 102520. 10.1016/j.jag.2021.102520

[B19] Kovacs G. Sowers N. (2018). Airway management in trauma. Emerg. Med. Clin. N. Am. 36, 61–84. 10.1016/j.emc.2017.08.006 29132582

[B20] Kushniruk A. Nohr C. Jensen S. Borycki E. M. (2013). From usability testing to clinical simulations: bringing context into the design and evaluation of usable and safe health information technologies: contribution of the IMIA human factors engineering for healthcare informatics working group. Yearb. Med. Inf. 22, 78–85. 10.1055/s-0038-1638836 23974552

[B21] Laput G. Brockmeyer E. Hudson S. E. Harrison C. (2015). “Acoustruments: passive, acoustically-driven, interactive controls for handheld devices,” in Proceedings of the 33rd annual ACM conference on human factors in computing systems CHI ’15 (New York, NY, USA: Association for Computing Machinery), 2161–2170. 10.1145/2702123.2702414

[B22] LeCun Y. Bengio Y. Hinton G. (2015). Deep learning. Nature 521, 436–444. 10.1038/nature14539 26017442

[B23] Lohser J. Slinger P. (2015). Lung injury after one-lung ventilation: a review of the pathophysiologic mechanisms affecting the ventilated and the collapsed lung. Anesth. Analgesia 121, 302–318. 10.1213/ANE.0000000000000808 26197368

[B24] Lu Y. H. Hsieh M. W. Tong Y. H. (1999). Unilateral vocal cord paralysis following endotracheal intubation--a case report. Acta Anaesthesiol. Sin. 37, 221–224. 10670122

[B25] McGaghie W. C. Issenberg S. B. Petrusa E. R. Scalese R. J. (2006). Effect of practice on standardised learning outcomes in simulation-based medical education. Med. Educ. 40, 792–797. 10.1111/j.1365-2929.2006.02528.x 16869926

[B26] Mesa-Cantillo C. M. Alonso-González I. G. Quintana-Suárez M. A. Ley-Bosch C. Ramírez-Casañas C. Sánchez-Medina J. J. (2023). A sound events detection and localization system based on YAMNet model and BLE beacons. Available at: https://accedacris.ulpgc.es/jspui/handle/10553/121504 (Accessed September 22, 2023).

[B27] Mitra A. Gave A. Coolahan K. Nguyen T. (2019). Confirmation of endotracheal tube placement using disposable fiberoptic bronchoscopy in the emergent setting. World J. Emerg. Med. 10, 210–214. 10.5847/wjem.j.1920-8642.2019.04.003 31534594 PMC6732169

[B28] Mort T. C. Keck J. P. Meisterling L. (2013). “Chapter 47 - endotracheal tube and respiratory care,” in Benumof and hagberg’s airway management. Editor Hagberg C. A. (Philadelphia: W.B. Saunders), 957–980.e5. 10.1016/B978-1-4377-2764-7.00047-6

[B29] Narendra R. Rajesh P. Manish M. Sudhir K. Gaur N. (2016). Airway management in ICU. Curr. Anesthesiol. Rep. 9, 116–127. 10.5005/jp/books/12670_3

[B30] Nejla T. Habiba B. S. A. Oussema J. Rim G. Nabil D. Mohamed F. H. (2017). Impact of monitoring endotracheal cuff pressure on endoscopic laryngo-tracheal injury: a randomized pilot study. J. Adv. Med. Med. Res. 24, 1–10. 10.9734/JAMMR/2017/37186

[B31] Pan S. J. Yang Q. (2010). A survey on transfer learning. IEEE Trans. Knowl. Data Eng. 22, 1345–1359. 10.1109/TKDE.2009.191

[B32] Pedregosa F. Varoquaux G. Gramfort A. Michel V. Thirion B. Grisel O. (2011). Scikit-learn: machine learning in Python. J. Mach. Learn. Res. 12, 2825–2830. 10.48550/arXiv.1201.0490

[B33] Salem M. R. Baraka A. S. (2013). “Chapter 32 - confirmation of endotracheal intubation,” in Benumof and hagberg’s airway management. Editor Hagberg C. A. (Philadelphia: W.B. Saunders), 657–682.e4. 10.1016/B978-1-4377-2764-7.00032-4

[B34] Samosky J. T. Baillargeon E. Bregman R. Brown A. Chaya A. Enders L. (2011). “Real-time ‘X-ray vision’ for healthcare simulation: an interactive projective overlay system to enhance intubation training and other procedural training,” in Medicine meets virtual reality 18 (Amsterdam, Netherlands (IOS Press)), 549–551. 10.3233/978-1-60750-706-2-549 21335854

[B35] Sprick C. Owen H. Hein C. Brown B. (2011). A new part task trainer for teaching and learning confirmation of endotracheal intubation. Stud. Health Technol. Inf. 163, 611–615. 10.3233/978-1-60750-706-2-611 21335866

[B36] Stewart S. L. Secrest J. A. Norwood B. R. Zachary R. (2003). A comparison of endotracheal tube cuff pressures using estimation techniques and direct intracuff measurement. AANA J. 71, 443–447. 15098531

[B37] Temko A. Nadeu C. (2006). Classification of acoustic events using SVM-based clustering schemes. Pattern Recognit. 39, 682–694. 10.1016/j.patcog.2005.11.005

[B38] Tikka T. Hilmi O. J. (2019). Upper airway tract complications of endotracheal intubation. Br. J. Hosp. Med. 80, 441–447. 10.12968/hmed.2019.80.8.441 31437047

[B39] Trueger N. S. (2013). “Airway management,” in Practical emergency resuscitation and critical care. Editors Lee J. Medlej K. Shah K. Weingart S. D. (Cambridge: Cambridge University Press), 17–27. 10.1017/CBO9781139523936.004

[B40] Tsalera E. Papadakis A. Samarakou M. (2021). Comparison of pre-trained CNNs for audio classification using transfer learning. J. Sens. Actuator Netw. 10, 72. 10.3390/jsan10040072

[B41] Tu H. N. Saidi N. Lieutaud T. Bensaid S. Menival V. Duvaldestin P. (1999). Nitrous oxide increases endotracheal cuff pressure and the incidence of tracheal lesions in anesthetized patients. Anesth. Analgesia 89, 187–190. 10.1213/00000539-199907000-00033 10389801

[B42] Varshney M. Sharma K. Kumar R. Varshney P. G. (2011). Appropriate depth of placement of oral endotracheal tube and its possible determinants in Indian adult patients. Indian J. Anaesth. 55, 488. 10.4103/0019-5049.89880 22174466 PMC3237149

[B43] Wiengand (2016). Procedure manual for high acuity, progressive, and critical care - E-book. Saunders. Philadelphia, Pennsylvania, United States. Elsevier Health Sciences.

