# Supplementary material for: Playing the pipes: acoustic sensing and machine learning for performance feedback during endotracheal intubation simulation
Source: Front Robot AI. 2023 Oct 30;10:1218174. doi: 10.3389/frobt.2023.1218174 (PMC10642916; doi:10.3389/frobt.2023.1218174)
Supplement: Supplementary file 1 [file DataSheet1.PDF]

Supplementary Material

Playing the Pipes: Acoustic Echoes and Machine Learning for Performance Feedback During Endotracheal Intubation Simulation

Authors

*** Correspondence:** Torjus Steffensen: torjus.l.steffensen@ntnu.no

1 SVM features

Three sets of features were tested for the support vector machine (SVM) classifier: FFT and basic features, MFCC, and wavelet scattering coefficients. Of the three, the set of FFT and basic features performed the best. Representative confusion matrices for the other two feature sets are presented in Supplementary Figure 1.

1.1 FFT and basic features

Audio samples were highpass filtered, normalized, and cut into 0.5 second chunksbefore the following features were extracted: zero crossing rate, root mean square, energy, spectral centroid, FFT magnitude. Supplementary Table 1 details regarding these features. The outputs were flattened to a 1 by 96378 array. PCA was used to reduce dimensionality by keeping the first 1000 principal components.

Supplementary Table 1: Basic feature list

Feature	Libraries	Code implementation	Dimension
Zero crossing rate	librosa	librosa.zero_crossing_rate(signal).sum()	1
2-norm	scipy	scipy.linalg.norm(signal)	1
Root mean square value	librosa	librosa.feature.rms(signal, sr).flatten()	1x188
Spectral centroid	librosa	librosa.feature.spectral_centroid(signal,sr)	1x188
FFT magnitude	numpy	numpy.abs(numpy.fft.fft(signal)).flatten()	1x96000

1.2 MFCC

Audio samples were cut into 0.5 second chunks, highpass filtered and normalize, and the following features were extracted: Mel-frequency Cepstral Coefficients (MFCC) were extracted using the mfcc function implemented in the librosa python library, returning 20 MFCCs per chunk.

1.3 Wavelet scattering coefficients

Wavelet scattering coefficients were extracted using the kymat.io python library. Audio was downsampled to 120 kHz, twice the highest frequency of the ultrasonic pulse. The samples were divided into 1 second chunks, and three levels of scattering coefficients were extracted using scaling factor 6 and 16 wavelets per octave.

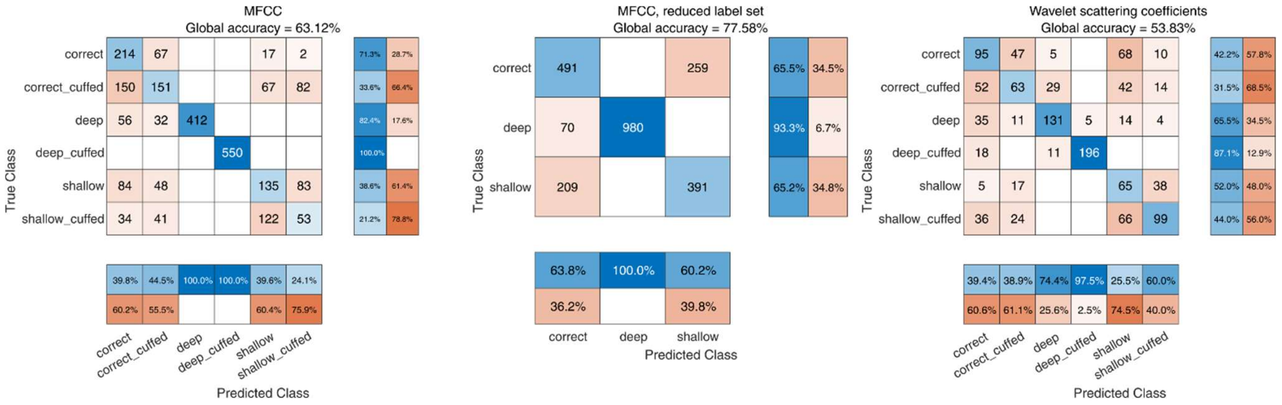

Supplementary Figure 2. Confusion matrices for the three additional feature sets. Left: SVM model trained on MFCC features using the full label set of intubation depth and cuff status. Centre: SVM model trained on the same features for the reduced label set of intubation depth only. Right: SVM model trained on wavelet scattering coefficients.

This is a provisional file, not the final typeset article
